# Supplementary material for: Complete Whole Genome Sequences of Escherichia coli Surrogate Strains and Comparison of Sequence Methods with Application to the Food Industry
Source: Microorganisms. 2021 Mar 16;9(3):608. doi: 10.3390/microorganisms9030608 (PMC8001026; doi:10.3390/microorganisms9030608)
Supplement: Supplementary file 1 [file microorganisms-09-00608-s001.pdf]

Figure S1. MinION Assemblies: Distribution of Contig Lengths. Depicts the size distribution of contigs based on their overall assembled lengths (bps) for each bacterial genomic assembly that was generated via the MinION assembler.

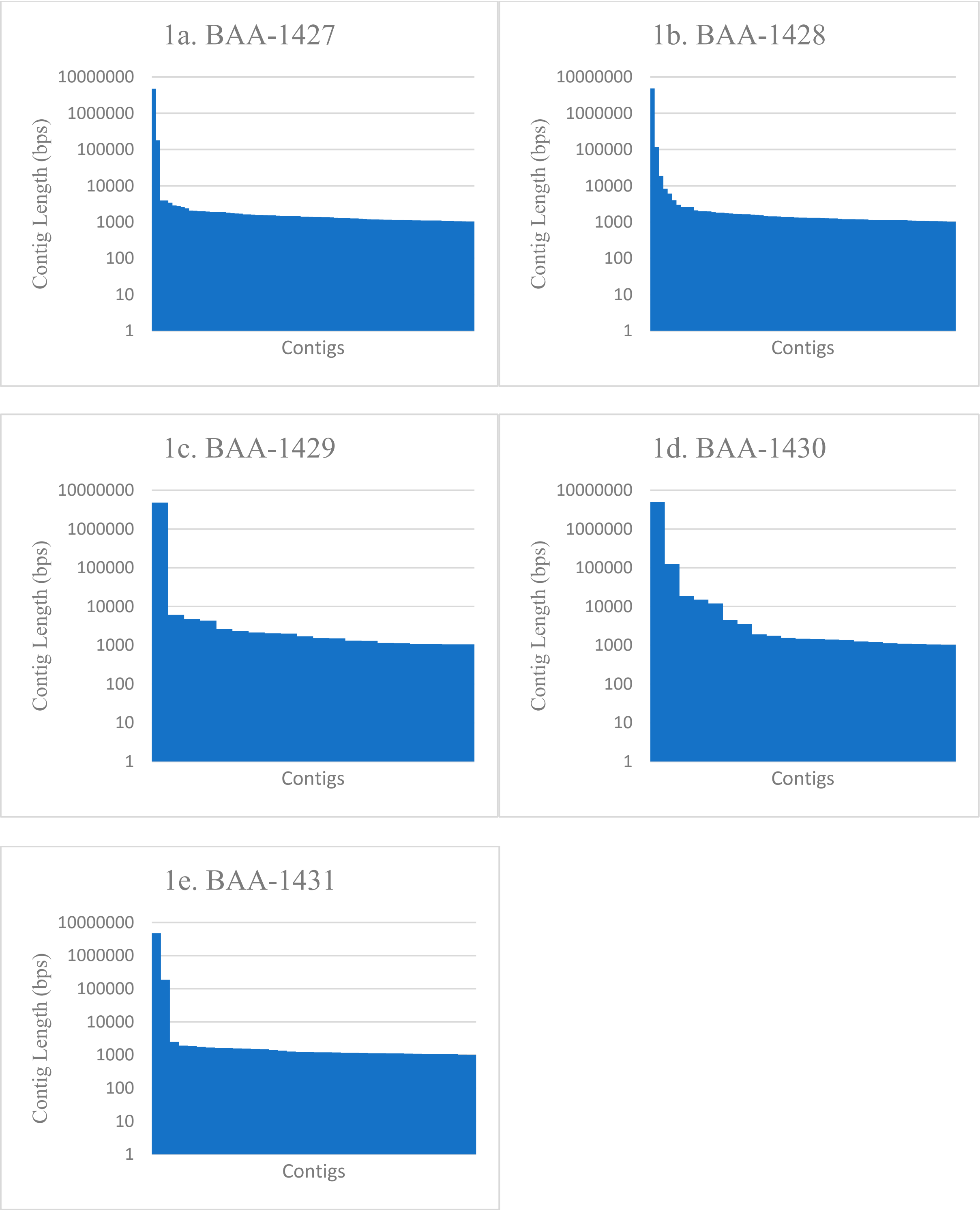

Figure S2. Miseq Assemblies: Distribution of Contig Lengths. Depicts the size distribution of contigs based on their overall assembled lengths (bps) for each bacterial genomic assembly that was generated via the MiSeq assembler.

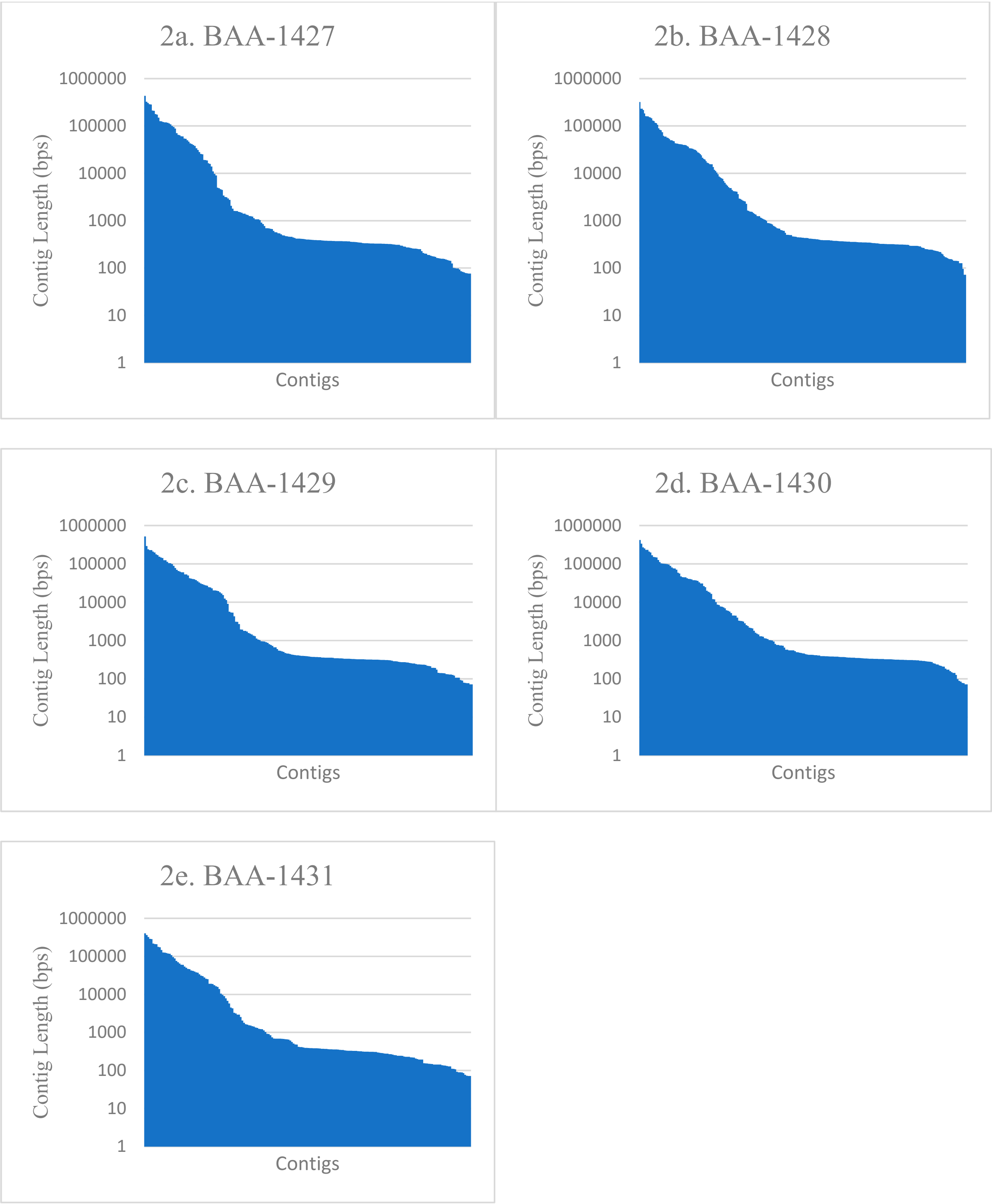

Table S1. List of virulence attributes examined.

Virulence Factor [1]	Related Gene	Pathogroup [2]	Pathogroup	NCBI Accession Number [3]
Afa/Dr family	*afaE-I, afaE-III, daaE, draE, draE2*	DAEC	Adherence	CAW30801, CAA54121, AAA23661, AAK16480, AAB65153
AAF's	*aafA, aafB, aafC, aafD, agg3A, agg3B, agg3C, agg3D, aggA, aggB, aggC, aggD, aggR*	EAEC	Adherence	AAB82330, AAD27809, AAD27810, AAD26595, AAM88298, AAM88297, AAM88296, AAM88295, AAA57454, AAA57453, AAA57452, AAA57451, CAA83535
Dispersin	*aap/aspU, aatA, aatB, aatC, aatD, aatP*	EAEC	Adherence	YP_006099176, YP_006099144, YP_006099145, YP_006099146, YP_006099147, YP_006099143
EAST-1	*astA*	EAEC	Toxin	BAA94855
Pet	*pet*	EAEC	Toxin	YP_006099165
Pic	*pic*	EAEC		YP_006098864
ShET1	*set1A, set1B*	EAEC	Toxin	YP_006098866, YP_006098865
ECP	*yagV/ecpE, yagW/ecpD, yagX/ecpC, yagY/ecpB, yagZ/ecpA, ykgK/ecpR*	EHEC	Adherence	NP_286006, NP_286007, NP_286008, NP_286009, NP_286010, NP_286011
Efa-1/LifA	*efa1*	EHEC	Adherence	AAD49229
Intimin	*eae*	EHEC	Adherence	NP_290259
Paa	*paa*	EHEC	Adherence	NP_287515
ToxB	*toxB*	EHEC	Adherence	YP_325655

Table S1 Continued

Chu	*chuA, chuS, chuT, chuU, chuW, chuX, chuY*	EHEC	Iron Uptake	NP_290080, NP_290079, NP_290082, NP_290086, NP_290083, NP_290084, NP_290085
EspP	*espP*	EHEC	Protease	YP_325580
StcE	*stcE*	EHEC	Protease	YP_325591
Ler	*ler*	EHEC	Regulation	NP_290288
Hemolysin	*hlyA, hlyB, hlyC, hlyD*	EHEC	Toxin	YP_325608, YP_325609, YP_325607, YP_325610
Stx (Shiga toxin)	*stx1A, stx1B, stx2A, stx2B*	EHEC	Toxin	NP_288673, NP_288672, NP_286976, NP_286977
Cif	*cif*	EHEC	Type III translocate protein	AAN07916
EspA, EspB, EspD, EspF, EspG, EspH	*espA, espB, espD, espF, espG, espH*	EHEC	Type III translocate protein	NP_290256, NP_290254, NP_290255, NP_290250, NP_290289, NP_290264
Map	*map*	EHEC	Type III translocate protein	NP_290262
NleA/EspI, NleC, NleD	*nleA/espI, nleC, nleD*	EHEC	Type III translocate protein	NP_287961, NP_286533, NP_286535
Tir	*tir*	EHEC	Type III translocate protein	NP_290261
IcsA (VirG)	*icsA/virG*	EIEC	Actin-based Mobility	NP_858315
LPS	*gtrA, gtrB, gtrII*	EIEC		NP_706257, NP_706258, NP_706259
Aerobactin	*iucA, iucB, iucC, iucD, iutA*	EIEC	Iron Uptake	NP_709454, NP_709455, NP_709456, NP_709457, NP_709458
Shu	*shuA, shuS, shuT, shuU, shuV, shuX, shuY*	EIEC	Iron Uptake	YP_405019, YP_405020, YP_405017, YP_405014, YP_405013, YP_405016, YP_405015
IcsP (SopA)	*icsP/sopA*	EIEC	Protease	NP_858404
Pic	*pic*	EIEC	Protease	NP_708747

Table S1 Continued

SigA	*sigA*	EIEC	Protease	NP_708742
T2SS (S. dysenteriae)	*gspC, gspD, gspE, gspF, gspG, gspH, gspI, gspJ, gspK, gspL, gspM*	EIEC	Secretion System	YP_404599, YP_404600, YP_404601, YP_404602, YP_404603, YP_404604, YP_404605, YP_404606, YP_404607, YP_404608, YP_404609
shET1, shET2	*set1A, set1B, senB*	EIEC	Toxin	YP_177614, YP_177613, YP_406304
Stx (S. dysenteriae (serotype 1))	*stxA, stxB*	EIEC	Toxin	YP_403025, YP_403026
BFP	*bfpA, bfpB, bfpC, bfpD, bfpE, bfpF, bfpG, bfpH, bfpI, bfpJ, bfpK, bfpL, bfpP, bfpU*	EPEC	Adherence	BAA84838, YP_002332159, YP_002332160, BAA84843, YP_002332163, BAA84845, YP_002332158, BAA84847, BAA84848, YP_002332168, BAA84850, YP_002332170, YP_002332165, BAA84842
Intimin	*eae*	EPEC	Adherence	AAC38392
Lymphostatin/ LifA	*lifA/efa1*	EPEC	Adherence	YP_002330705
Paa	*paa*	EPEC	Adherence	YP_006158657
EspC	*espC*	EPEC	Protease	AAG37043
Ler	*ler*	EPEC	Regulation	AAC38364
Per	*perA/bfpT, perB/bfpV, perC/bfpW*	EPEC	Regulation	BAA84859, BAA84860, BAA84861
CDT	*cdtA, cdtB, cdtC*	EPEC	Toxin	CAD48849, CAD48850, CAD48851
EAST1	*east1*	EPEC	Toxin	AHY03744
Cif	*cif*	EPEC	Type III translocate protein	PRJEA32571

Table S1 Continued

EspA, EspB, EspD, EspF, EspG, EspH	*espA, espB, espD, espF, espG, espH*	EPEC	Type III translocate protein	AAC38394, AAC38396, AAC38395, AAC38400, AAC38363, AAC38387
Map	*orf19*	EPEC	Type III translocate protein	AAC38389
NleA/EspI, NleC, NleD	*nleA, nleC, nleD*	EPEC	Type III translocate protein	EIQ72111, YP_003498583, YP_003498587
Tir	*tir*	EPEC	Type III translocate protein	AAC38390
Adhesive fimbriae	*cfaB, cofA, cooA, cs3, csbA, cseA, csfA, csnA, cssA, csvA, cswA*	ETEC	Adherence	AAC41415, BAB62897, AAT07441, AAA23614, AAS89777, AAD30557, CAA11820, AAL31637, AAC45093, AAK09045, AAK09047
EtpA	*etpA, etpB*	ETEC	Adherence	YP_006203830, CBJ04459
Heat-labile toxin (LT)	*eltA, eltB*	ETEC	Toxin	AAA24685, AAA98064
Heat-stable toxin (ST)	*estIa*	ETEC	Toxin	YP_003294006
FdeC	*fdeC*	NMEC	Adherence	YP_002390132
S fimbriae	*sfaA, sfaB, sfaC, sfaD, sfaE, sfaF, sfaG, sfaH, sfaS*	NMEC	Adherence	YP_006100306, YP_006100305, YP_006100304, YP_006100307, YP_006100308, YP_006100309, YP_006100310, YP_006100312, YP_006100311
AslA	*aslA*	NMEC	Invasion	AAG10151
Ibes	*ibeA, ibeB, ibeC*	NMEC	Invasion	AAF98391, AAD30205, AAD28716
K1 capsule	*kpsD, kpsM, kpsT*	NMEC	Invasion	AAA21682, AAA24046, AAA24047
OmpA	*ompA*	NMEC	Invasion	AAF37887
TraJ	*traJ*	NMEC	Invasion	AAA92657

Table S1 Continued

CNF-1	*cnf1*	NMEC	Toxin	CAA50007
Dr adhesins	*draA, drab, draC, draD, draE, draP*	UPEC	Adherence	AAK16475, AAK16476, AAK16477, AAK16478, AAK16480, AAK16479
F1C fimbriae	*focA, focC, focD, focF, focG, focH focI*	UPEC	Adherence	NP_753153, NP_753155, NP_753156, NP_753157, NP_753158, NP_753159, NP_753154
P fimbriae	*papA, papA, papB, papB, papC, papC, papD, papD, papE, papE, papF, papF, papG, papG, papH, papH, papI, papI, papJ, papJ, papK, papK*	UPEC	Adherence	NP_755467, NP_757036, -, -, NP_755465, NP_757034, NP_755464, NP_757033, NP_755460, NP_757029, NP_755459, NP_757028, NP_755458, NP_757027, NP_755466, NP_757035, NP_755468, NP_757037, NP_755463, NP_757032, NP_755461, NP_757030
S fimbriae	*sfaA, sfaB, sfaC, sfaD, sfaE, sfaF, sfaG, sfaH, sfaS, sfaX, sfaY*	UPEC	Adherence	YP_540124, YP_540123, YP_540122, YP_540125, YP_540126, YP_540127, YP_540128, YP_540130, YP_540129, YP_540132, YP_540131
Type 1 fimbriae	*fimA, fimB, fimC, fimD, fimE, fimF, fimG, fimH, fimI*	UPEC	Adherence	NP_757241, NP_757239, NP_757243, NP_757244, NP_757240, NP_757245, NP_757247, NP_757248, NP_757242
TcpC	*tcpC*	UPEC	Immune Evasion	NP_754290
Aerobactin	*iucA, iucB, iucC, iucD, iutA*	UPEC	Iron Uptake	NP_755502, NP_755501, NP_755500, NP_755499, NP_755498
Chu	*chuA, chuS, chuT, chuU, chuV, chuW, chuX, chuY*	UPEC	Iron Uptake	NP_756170, NP_756169, NP_756175, NP_756179, NP_756180, NP_756176, NP_756177, NP_756178

Table S1 Continued

Enterobactin	*entA, entB, entC, entD, entE, entF, fepA, fepB, fepC, fepD, fepE, fepG*	UPEC	Iron Uptake	NP_752614, NP_752613, NP_752611, NP_752599, NP_752612, NP_752604, NP_752600, NP_752610, NP_752606, NP_752608, NP_752605, NP_752607
IroN	*iroN*	UPEC	Iron Uptake	NP_753164
Pic	*pic*	UPEC	Protease	NP_752289
Sat	*sat*	UPEC	Protease	NP_755494
Tsh	*vat*	UPEC	Protease	NP_752330
CNF-1	*cnf1*	UPEC	Toxin	YP_543855
Hemolysin	*hlyA, hlyB, hlyC hlyD*	UPEC	Toxin	NP_755445, NP_755448, NP_755444, NP_755449

[1] Table was constructed from pre-existing E. coli data located on the VFDB [52-55]

[2] Diffusely adherent *E. coli* (DAEC), Enteroaggregative *E. coli* (EAEC), Enterohemorrhagic *E. coli* (EHEC), Enteroinvasive *E. coli* (EIEC), Enteropathogenic *E. coli* (EPEC), Enterotoxigenic *E. coli* (ETEC), Neonatal meningitis-associate *E. coli* (NMEC), Uropathogenic *E. coli* (UPEC).

[3] NCBI accession numbers are listed in the same order as the related gene they are associated with. [52-55]

Table S2. Comprehensive list of alterations within the *rpoB* genes of each sample and assembly type for each virulence factor screened.

Assembly	Sample	Codon [1]	Mutation	Alteration	Amino Acid Shift
MiSEQ	1427	206*	Transition	GCG → GCA	A to A
	1428	486*	Transition	ACC → ACT	T to T
	1430	489*	Transversion	CCA → CCT	P to P
	1430	623*	Transition	TTG → CTG	L to L
	1430	846*	Transversion	GGT → GGG	G to G
MINION	1427	9	Deletion	AAA → AA-	-
	1427	115	Deletion	AAA → AA-	-
	1427	206*	Transition	GCG → GCA	A to A
	1427	265	Deletion	AAA → AA-	-
	1427	319	Deletion	CTG → C-G	-
	1427	431	Deletion	AAG → A-G	-
	1427	468	Transition	CTG → CCG	L to P
	1427	618	Deletion	CAG → C-G	-
	1427	643	Deletion	CTG → C-G	-
	1427	671	Transition	CTG → CCG	L to P
	1427	941	Deletion	AAA → AA-	-
	1427	1117	Deletion	CCT → C--	-
	1427	1238	Deletion	CTG → --G	-
	1428	8	Deletion	AAA → AA-	-

Table S2 Continued

1428	83	Deletion	CAG → C-G	-
1428	114	Deletion	GTA → GT-	-
1428	226	Deletion	GAA → G-A	-
1428	264	Deletion	GAA → G-A	-
1428	278	Deletion	GAA → G-A	-
1428	319	Deletion	CTG → C-G	-
1428	372	Deletion	CCT → CC-	-
1428	468	Deletion	CTG → C-G	-
1428	486*	Transition	ACC → ACT	A to A
1428	563	Deletion	ACC → A-C	-
1428	618	Transition	CAG → CGG	Q to R
1428	643	Deletion	CTG → C-G	-
1428	649	Transition	CAG → CGG	Q to R
1428	671	Deletion	CTG → C-G	-
1428	940	Deletion	GAA → G-A	-
1428	987	Deletion	GAG → GA-	-
1428	1038	Transition	CAG → CGG	Q to R
1428	1062	Transition	CCT → CCC	R to P
1428	1117	Deletion	CTG → C-G	-
1428	1238	Deletion	CTG → --G	-

Table S2 Continued

1430	4	Transition	TCC → CTC	S to L
1430	8	Deletion	AAA → -AA	-
1430	83	Deletion	CAG → C--	-
1430	112	Transition	GGC → AGC	G to S
1430	113	Deletion	AAC → AA-	-
1430	114	Deletion	GGT → --T	-
1430	115	Deletion	ACC → -CC	-
1430	183	Transition	TGG → CGG	W to R
1430	227	Deletion	AAA → AA-	-
1430	265	Deletion	AAA → AA-	-
1430	319	Deletion	CTG → C-G	-
1430	372	Deletion	CCT → C-G	-
1430	431	Deletion	AAG → A-G	-
1430	468	Transition	CTG → CCG	L to P
1430	489*	Transversion	CCA → CCT	P to P
1430	535	Deletion	CCA → CC-	-
1430	618	Transition	CAG → CGG	Q to R
1430	623*	Transition	TTG → CCG	L to P
1430	633	Deletion	CTG → C-G	-
1430	846*	Transversion	GGT → GGG	G to G

Table S2 Continued

	1430	1038	Deletion	CAG → C-G	-
	1430	1062	Transition	CCT → CCC	P to P
	1430	1117	Deletion	CTG → C--	-
	1430	1238	Deletion	CTG → C-G	-
	1430	1253	Transition	CTG → CCG	L to P
Hybrid	1427	206*	Transition	GCG → GCA	A to A
	1428	486*	Transition	ACC → ACT	T to T
	1430	489*	Transversion	CCA → CCT	P to P
	1430	623*	Transition	TTG → CTG	L to L
	1430	846*	Transversion	GGT → GGG	G to G
Rif-Mutant	1427	206*	Transition	GCG → GCA	A to A
	1427	533R	Transition	CTC → CCC	L to P
	1428	486*	Transition	ACC → ACT	T to T
	1428	512R	Transition	TCT → CCT	S to P
	1430	489*	Transversion	CCA → CCT	P to P
	1430	526R	Transition	CAC → TAC	H to Y
	1430	623*	Transition	TTG → CTG	L to L
	1430	846*	Transversion	GGT → GGG	G to G

[1] Codon number within the rpoB gene of the *E. coli* str. K-12 substr. MG1655 (NC_000913.3) reference strain.

* Indicates that this mutation was found within all four assemblies.
R Documented to confer Rif-resistance.
